# Longitudinal effects of direct observation of hand hygiene practices and monitoring of alcohol-based handrub consumption

**DOI:** 10.1017/ash.2023.322

**Published:** 2023-09-29

**Authors:** Retsu Fujita, Rika Yoshida, Satoshi Hori

## Abstract

**Background:** In healthcare facilities, hand hygiene is important for infection control. The WHO recommends monitoring the consumption of alcohol-based handrub (ABHR) and direct observation of hand hygiene practices to ensure compliance with hand hygiene practices. Monitoring of ABHR is widely used, but direct observation is not widely performed, particularly in small facilities and non–acute-care facilities. We evaluated the effects of direct observation of hand hygiene practices and monitoring of ABHR consumption, with feedback to staff, on ABHR consumption and hand hygiene compliance. **Methods:** We conducted a prospective intervention study over a 4-year period. Monitoring of ABHR consumption and direct observation of hand hygiene practices, with periodic feedback to staff, was implemented in 17 facilities of varying types: 5 large-scale acute-care facilities, 6 middle-to-small-scale acute-care facilities, and 6 non–acute-care facilities. Statistics for ABHR consumption were calculated before and after the implementation of direct observation of hand hygiene practices, and the change in ABHR consumption was calculated. The paired *t* test was used to assess the statistical significance of changes. A generalized linear mixed model analysis was performed to assess factors associated with ABHR consumption. **Results:** The total observation time was 1,225 months (625 months before direct observation, 600 months after direct observation), and the average observation time per facility was 36.0 months (± 27.5). All facilities implemented ABHR consumption monitoring within 1 month of starting the study. However, the mean time required to implement direct observation of hand hygiene practices was 24.7 (±19.1) months. There was a significant increase in ABHR consumption in large and middle-to-small-scale acute-care facilities (*P* < .0001) after implementing the direct observation. However, there was not a significant increase for ABHR consumption in non–acute-care facilities (*P* = .14). Multivariable regression analysis showed that the hospital ward type, duration of ABHR consumption monitoring, and duration of direct observation of hand hygiene practices were independently associated with ABHR consumption. **Conclusions:** ABHR consumption increased in all facilities that implemented direct observation, but the change was not statistically significant in non–acute-care facilities. The generalized linear mixed model analysis showed significant associations between ABHR consumption and hospital ward type and time to monitoring of ABHR consumption and direct observation of hand hygiene practices. Direct observation of hand hygiene practices should be implemented more widely. The effect of intervention intensity should be evaluated in future studies.

**Disclosures:** None

